# Comparing different intensities of active referral to smoking cessation services in promoting smoking cessation among community smokers: a study protocol of a cluster randomized controlled trial

**DOI:** 10.1186/s12889-018-5782-1

**Published:** 2018-07-04

**Authors:** Xue Weng, Man Ping Wang, Yi Nam Suen, William Ho Cheung Li, Yongda Wu, Derek Yee Tak Cheung, Antonio Cho Shing Kwong, Vienna Wai Yin Lai, Sophia Siu Chee Chan, Tai Hing Lam

**Affiliations:** 1 School of Nursing, University of Hong Kong, 4/F, William MW Block, 21 Sassoon Road, Pokfulam, Hong Kong SAR; 2 Department of Psychiatry, University of Hong Kong, 2/F, New Clinical Building, Queen Mary Hospital, 102 Pokfulam Road, Pokfulam, Hong Kong SAR; 3 Hong Kong Council on Smoking and Health, Unit 44-2-03, 44/F, Hopewell Centre, 183 Queen’s Road East, Wanchai, Hong Kong SAR; 4 School of Public Health, University of Hong Kong, G/F, Patrick Manson Building (North Wing), 7 Sassoon Road, Pokfulam, Hong Kong SAR

**Keywords:** Smoking cessation, Active referral, Community smoker, Randomized controlled trial

## Abstract

**Background:**

Actively referring smokers to smoking cessation (SC) services could increase quitting and is scalable for the population. The objective of this study is to compare 2 different intensities of SC active referral for smokers in the community of Hong Kong.

**Methods/design:**

This is a single-blind, parallel 3-armed cluster randomized controlled trial (cRCT) consisting of high-intensity SC active referral (HAR Group), low-intensity SC referral by text messaging on promoting SC services use (Text Group) and a control group receives general very brief advice. Biochemically validated daily smokers will be proactively recruited in the community from 68 clusters (recruitment sessions). The primary outcome is self-reported 7-days point prevalence abstinence (PPA) at the 3- and 6- month follow-ups. Secondary outcomes are SC service use, smoking reduction rate (SRR, daily cigarette consumption reduced by ≥50%; excluding quitters) and biochemically validated quit rate (exhaled CO < 4 ppm and salivary cotinine < 10 ng/ml). Outcome assessors and data analysts will be blinded to group allocation. Intention-to-treat principle and Generalized Estimating Equation (GEE) regressions will be used for data analysis.

**Discussion:**

This will be the first trial on evaluating the efficacy of the 2 different intensities of SC active referral on smoking cessation in community smokers. It is anticipated that the results from this trial can provide evidence to the effectiveness of high-intensity active referral to SC services and low intensity SC referral by using text messaging in achieving smoking abstinence.

**Trial registration:**

ClinicalTrials.gov Identifier: NCT02804880, June 17, 2016.

## Background

Despite decades of public health education and interventions on smoking cessation (SC), tobacco use remains the leading cause of preventable death causing over 7 million deaths worldwide every year [1]. Recent data indicates a decrease in the overall smoking prevalence but still with around 615,000 daily cigarette smokers in Hong Kong in 2017 [2]. Evidence has shown that smokers received SC services increases successful rate in quitting [3–6]. Although a variety of free SC services (e.g. quit-lines, clinics) are available in Hong Kong, the utility rate is low and only 14.2% of smokers had ever used [2]. Low-cost, easy accessibility and effective SC services are warranted to provide advice, medication, and support for people to quit smoking [7].

Referring smokers to SC services is one of the cheap and scalable strategies that can maximize usage of public cessation counselling and treatment. Most of current SC services depend on passive referral strategies that require smokers to seek assistance on their own, such as calling the quit-line or attending the SC clinics [8]. Unlike passive referrals that rely on smokers’ self-initiation, active referral emphasizes physicians or other healthcare professionals efforts to refer smokers to SC services directly [9, 10]. Allowance for smokers to choose their preferred service providers and cessation methods might also improve smokers’ engagement in the service and hence enhance the outcomes [11]. Proactive referral approach can help overcome the barrier of self-initiation as most community smokers quit on their own without SC services [12]. Our previous randomized controlled trial (RCT) showed that moderate active referral of SC services significantly increased smoking abstinence rate at 6 months when compared with brief general SC advice (17.2% vs. 11.5%, *p* = 0.02) [11].

Comparing with traditional telephone cessation assistance, interventions delivered via text messaging are more convenient and accessible, which could potentially extend the reach to SC services [4] and promote SC [13–15]. A recent meta-analysis of 15,593 smokers (20 RCTs) showed that the overall odds of smoking abstinence in text messaging group were 1.37 times higher than the control group [16]. The intensity of interventions in previous RCTs varies, in terms of the frequency, intensity and duration of text messaging in the intervention period, the degree and methods for personalization and tailoring of the text messaging. Substantial evidence has shown that intensive interventions produce more abstinence than less intensive interventions in the clinical setting [4]. Therefore, it is assumed that active referral intervention delivered via high-intensity text messaging will also result in more abstinence when compared with low-intensity text messaging. This study aims to evaluate the effectiveness of 2 different intensities of active referral interventions: (1) high-intensity active referral to SC services and (2) low-intensity SC service referral by using text messaging. The control group will receive general brief SC advice.

## Methods/design

### Overview of design

**Fig. 1:**
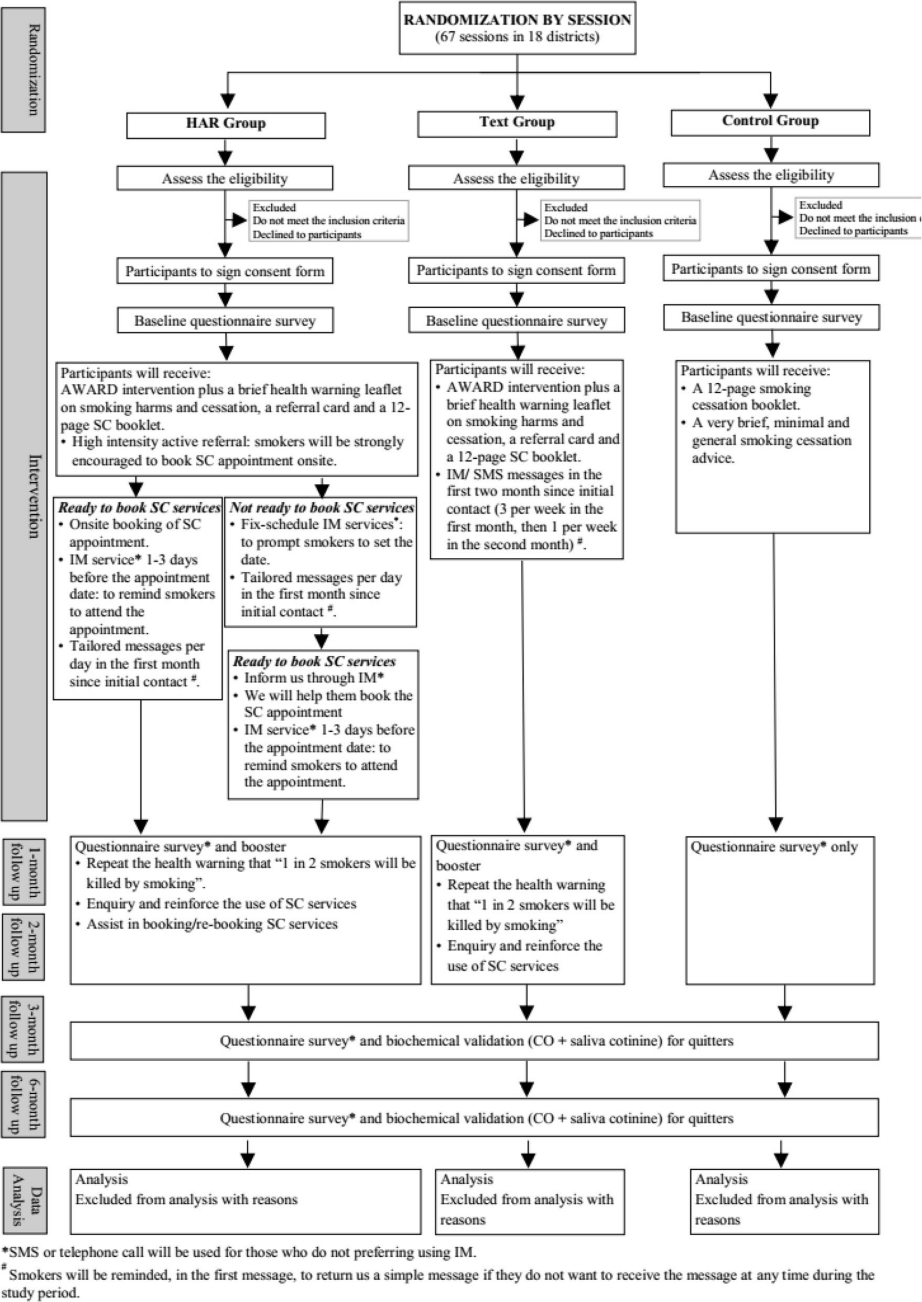
Study design

This is a single-blinded, parallel 3-armed cluster RCT (cRCT) on 1200 smokers participated in the Hong Kong 7th “Quit to Win” (QTW) Contest conducted in 2016–17. The QTW Contest [11, 17–20] is organized by the Hong Kong Council on Smoking and Health (COSH) each year since 2009 to promote SC in the general community. Based on the recruitment sessions, participants will be randomly assigned to 1 of the following 3 conditions: (1) high intensity active referral to SC services (HAR Group); (2) low intensity SC service referral using text messaging on promoting and encouraging SC service use (Text Group); or (3) general brief SC advice (Control Group). All participants will receive a 12-page self-help SC booklet at baseline. HAR Group and Text Group will also receive AWARD model guided SC advice with a warning leaflet and a referral card. Control Group will only receive general brief advice at baseline. The trial will follow the Consolidated Standards of Reporting Trials (CONSORT) criteria for the design [21] and is shown in Fig. 1.

### Participants

Inclusion criteria:

- Hong Kong residents aged 18 or above;
- Currently smoking at least 1 cigarette per day in the past 3 months;
- Able to communicate in Cantonese (and read Chinese);
- Exhaled carbon monoxide (CO) 4 ppm or above as validated by CO Smokerlyzer;
- Intend to quit or reduce smoking [22];
- Having a local phone number for follow-up.

Exclusion criteria:

- Having physical or cognitive difficulties in communication;
- Currently following other SC programs.

### Recruitment

A total of 68 recruitment activities will be organized in all 18 Hong Kong districts aiming to recruit 1200 participants over 3 months. University students majoring in nursing, public health and related fields and volunteers of non-governmental organizations (NGOs) will be trained as SC ambassadors in a two-day workshop (8 h each) for onsite recruitment. To reach as many as eligible smokers, booths containing SC and recruitment information will be set up in shopping malls and public areas. Using persuasive techniques such as the ‘foot-in-the-door’ method [23], SC ambassadors will ask smokers simple questions (e.g., daily cigarette consumption, history of smoking, general health status) to arouse their interest in participating in the Contest. Informed consent for voluntary participation will be obtained from the eligible participants before administering the baseline questionnaire.

### Randomization and blinding

cRCT design will be used in which all participants will be randomly assigned to 1 of the 3 groups based on the recruitment session. The randomization of group assignment will be generated before recruitment. One investigator who does not participate in recruitment will randomly generate blocks, with each block size equal to 3, 6 or 9, containing a random permutation of the 3 RCT groups. The numbers for the permutation in the blocks will be generated from the website http://www.random.org (a website for generating random integers), and then merge with the list of all recruitment days. As the intervention cannot be completely blinded in this study, the RCT is single-blinded that all outcome assessors and data analysts will be blinded to group allocation.

### Sample size and power analysis

The computer program G*Power is used to calculate the sample size [24]. The proposed sample size is based on the primary outcome of self-reported 7-day point prevalence of abstinence (PPA) at 3 months. Based on the QTW study in 2015, the 3-month PPA for the moderate-intensity active referral intervention and the control group was 18.7 and 14%, respectively [11]. Therefore, the effect size (odds ratio) for the intervention in the present study is set conservatively at 1.33. To detect a significant difference in quit rate with a power of 80% and a significance level of 5%, 971 participants in each arm are required. Given the limited resources and recruitment period, the maximum total sample size is 1200 (400 per group). The *p*-value and the power for testing the expected effect size (1.54) will be 0.08 and 57.3%.

#### Interventions

Each RCT group consists of one or a combination of the following components as the intervention. The detail is shown in Table 1.

**Table 1:** Summary of intervention in 3 groups

	HAR Group	Text Group	Control Group
Onsite active referral + tailored messages	✓		
Text messaging		✓	
AWARD advice + health warning leaflet + referral card + self-help booklet	✓	✓	
General brief advice + self-help booklet			✓

#### On-site active referral for HAR group

Interventions in HAR group are designed to promote the use of the existing SC services to increase abstinence. Five major cessation services in Hong Kong will be involved. They had successful collaboration with the Contest in 2015, and their characteristics were reported previously [11, 25]. Well-trained SC ambassadors will assist on-site booking for SC services based on participants’ preferred services providers and available time. For participants who are not ready to seek SC services at on-site, they will be encouraged to set a date for appointment booking. SC ambassadors will follow them through the telephone at 1 week. They can also inform us through instant messages or telephone calls anytime between 1-week and 1-month. SC ambassadors will help participants book the appointment once receiving their decisions on SC services providers, preferred time and clinics.

#### Tailored messages for HAR group

Tailored, automatic, and fix-schedule messages will be sent once per day for the first month (or stop upon request by the smokers) via instant messaging (IM) services (e.g., WhatsApp, WeChat) since initial contact. For smokers who do not use or refuse to receive IM messages, they will be contacted via short messaging service (SMS) or telephone calls. Contents of the tailored messages will be developed through a qualitative study using open-ended questions in participants of QTW 2014. Tailored messages will include (1) brief health warning, (2) benefits of quitting, (3) SC services and their effectiveness (4) story about pleasant experiences and successful quitting of smokers in the past, and (5) readiness to make and be adherent to SC appointments.

Details of successfully booked SC appointment, such as SC services address, contact information, date, and appointment number will be delivered to the smokers using messages or telephone calls. All smokers will receive a reminder-to-attend message or telephone call 1–3 days before the appointment date. Research staff will monitor the use of SC services by smokers at each follow-up (1-, 2-, 3- and 6-months) and assist participants to book or re-book the appointments if necessary. We shall liaise with the existing service providers and seek their assistance in helping the smokers in a timely manner.

#### Text messaging for Text group

The text-messaging intervention aims to motivate smokers to book the SC appointments by themselves. They will be introduced (using the referral card) and motivated to use the SC services at onsite. IM/SMS messages (3 per week in the first month, then 1 per week in the second month) will be sent to encourage them to book an SC appointment since initial contact. A total of 16 messages will be sent to the smokers. The messages will be simpler and more generic than the messages in HAR group. Research staff will monitor the SC services use at each follow-up (1-, 2-, 3- and 6-months) and encourage smokers to book or re-book the appointment if necessary. This is a much cheaper and brief method than HAR group.

#### AWARD advice for HAR and Text groups

AWARD model-guided advice will be delivered to smokers onsite, which include (1) ask about smoking history, (2) warn the subject about the increased risk of premature death (with a brief health warning leaflet, see below for information), (3) advise the subject to quit immediately, (4) refer the subject to existing cessation services (with a referral card, see below for information), and (5) do it again if the subject fails to quit. The whole process of AWARD can be delivered within 30 s to 1 min. The model has been validated in our previous trials [11, 17–20].

#### Self-help materials

Following the previous trial in 6th QTW in 2015 [25], the 2-side health warning leaflet, which systematically covers the most important messages to motivate SC, as well as the pocket size referral card will be disseminated to the smokers in the 2 intervention groups. The content of referral card includes brief information and highlights of existing SC services, contact details, available therapies, and incentive information.

All participants in 3 groups will receive a 12-page self-help booklet developed by the COSH. The content includes information about benefits of quitting, smoking and diseases, methods to quit, how to handle withdrawal symptoms, declaration of quitting, etc.

#### General brief advice for Control group

Participants in the control group will receive very brief, minimal and general SC advice, such as “Please quit smoking for improving health and save money”, “Please refer to the self-help booklet for the details about smoking cessation” and “Please call us if you have any enquiry”.

### Data collection

#### Baseline

The baseline questionnaire data includes four parts and will be collected by trained SC ambassadors. The first part measures participants’ smoking status (e.g., average number of cigarettes smoked per day, the age of starting smoking and the usual time having the first cigarette smoked each day, attempts to quit or reduce, methods used in past quitting attempts, etc.). The second part measures participants’ readiness to quit, and perceived importance, difficulties and confidence to quit smoking. The third part asks participants’ knowledge about smoking (e.g., e-cigarette and risk of smoking). The final part collects sociodemographic data (e.g., sex, age, education level, number of children, occupation, marital status, annual income, etc.).

#### Follow-up

The follow-up surveys will be conducted by telephone at 1, 2, 3 and 6 months after the baseline. A set of questionnaire similar to the baseline questionnaire will be used to collect information on smoking behavior, quit attempts, smoking-related psychological factors and perceived social support in the quitting process, and the use of referred SC services. Participants who reported 7-day PPA at 3 and 6 months will be invited for a biochemical validation using the exhaled carbon monoxide (CO) and saliva cotinine tests.

#### Outcome assessments

The primary outcome is self-reported 7-day PPA at 3 and 6 months.

Secondary outcomes include:

1. SC services use: calling a hotline of the SC services, booking an appointment, SC clinic attendance, counselling session attendance, and other indicators to be further specified after liaison with the existing SC services (e.g. services providers’ records on services utilization). The number of referrals will be calculated for the baseline and 1-, 2-, 3- and 6-month follow-ups.
2. Biochemically-verified past 7-day PPA using exhaled-air CO levels < 4 ppm and saliva cotinine concentration < 10 ng/ml [26, 27].
3. Smoking reduction is defined as daily cigarette consumption reduction by > 50% compared with baseline.

### Data analysis

Data will be entered into SPSS for Windows (version 23). Descriptive statistics such as frequency, percentage, and mean will be used to summarise the outcomes and other variables. The main analysis will be a comparison between both intervention groups and the control group for the proportions of smoking abstinence at 3 and 6 months, using chi-square tests and odds ratios with 95% confidence intervals. Generalized Estimating Equation (GEE) models will be applied to test the intervention effect, to identify the baseline predictors of successful quitting and to assess the changes in smoking-related factors over time. Intention-to-treat (ITT) analysis will be used in which participants who lose contact or drop out in the follow-ups will be treated as a failure to achieve any cessation outcome. Multiple imputations will be used to compute missing data for outcome variables.

## Discussion

To the best of our knowledge, this is the first RCT conducted to evaluate whether high-intensity active referral to SC services is more effective than low intensity of SC referral by using text messaging in achieving abstinence among smokers in the community. Since the efficiency of active referral of SC services (moderate level of SC active referral by collecting smokers’ details and send to SC providers for follow-up) has been proved in the previous trial [11], the present study explores further by comparing the efficacy of the 2 different intensities of SC active referral. We also expect that active referral with high intensity will achieve high rates on smoking abstinence and smoking reduction by comparing the effectiveness of HAR, moderate level SC active referral and text-messaging referral in a 2-year combined model.

This study has several significant strengths. Firstly, most SC service referral studies were conducted in the health care settings [10, 28–30]; research conducted in the community settings is scarce [11]. The study fills this gap by providing an active referral intervention for community smokers who are not usually self-initiated to seek SC assistance. Secondly, follow-up assessments will be carried out at 1, 2, 3 and 6 months which allows us to keep longitudinal tracking on the effects of the active referral interventions on abstinence and changes in smoking-related psychosocial factors. Thirdly, the intervention approaches in this study (e.g., on-site active referral, text messaging, AWARD advice, referral card, self-help booklet) are brief and flexible. They can reach large numbers of community smokers at relatively low cost [11]. Therefore, the findings obtained will have significant implications for SC practice. Finally, the study will also have important implications for current SC policy, given the low utilization of SC services and a large number of cigarette smokers. If the intervention is effective in helping smokers to quit, our findings will address the active role of community health workers and SC service providers in promoting SC in the community.

## Abbreviations

AWARD: Ask, Warn, Advice, Refer and Do-it-again
CO: Carbon monoxide
CONSORT: Consolidated Standards of Reporting Trials
COSH: Hong Kong Council on Smoking and Health
GEE: Generalized Estimating Equation
HAR: High-intensity active referral
IM: Instant messaging
ITT: Intention-to-treat
NGOs: Non-governmental organizations
PPA: Point prevalence abstinence
QTW: Quit to Win
RCT: Randomized controlled trial
SC: Smoking cessation
SMS: Short messaging service
